# Shaping Information Processing: The Role of Oscillatory Dynamics in a Working Memory Task

**DOI:** 10.1523/ENEURO.0489-21.2022

**Published:** 2022-09-13

**Authors:** Hesham A. ElShafei, Ying Joey Zhou, Saskia Haegens

**Affiliations:** 1 Donders Institute for Brain, Cognition, and Behaviour, Radboud University, 6525 EN, Nijmegen, The Netherlands; 2 Department of Psychiatry, Columbia University, New York, New York 10032; 3 Division of Systems Neuroscience, New York State Psychiatric Institute, New York, New York 10032

**Keywords:** alpha rhythm, beta rhythm, brain oscillations, MEG, working memory

## Abstract

Neural oscillations are thought to reflect low-level operations that can be used for higher-level cognitive functions. Here, we investigated the role of brain rhythms in the 1–30 Hz range by recording MEG in human participants performing a visual delayed match-to-sample paradigm in which orientation or spatial frequency of sample and probe gratings had to be matched. A cue occurring before or after sample presentation indicated the to-be-matched feature. We demonstrate that alpha/beta power decrease tracks the presentation of the informative cue and indexes faster responses. Moreover, these faster responses coincided with an augmented phase alignment of slow oscillations, as well as phase–amplitude coupling between slow and fast oscillations. Importantly, stimulus decodability was boosted by both low alpha power and high beta power. In summary, we provide support for a comprehensive framework in which different rhythms play specific roles: slow rhythms control input sampling, while alpha (and beta) gates the information flow, beta recruits task-relevant circuits, and the timing of faster oscillations is controlled by slower ones.

## Significance Statement

Brain oscillations reflect low-level operations, building blocks, that control the flow of information through the brain. We propose and test a novel comprehensive framework in which slow oscillations control input sampling, alpha oscillation gates information flow, beta oscillation recruits task-relevant circuits, and the timing of faster oscillations is controlled by slower ones. We collected MEG data while participants performed a visual delayed match-to-sample task with precues and retro-cues. Phase alignment of slow oscillations, governing input sampling, indexed faster responses. Alpha/beta power, gating information flow, boosted behavior and tracked informative cues. Low-alpha (gating) and high-beta (circuit setup) power boosted signal information content. This is an essential step toward a more unified framework regarding the role of oscillatory dynamics in shaping information processing.

## Introduction

Brain oscillations reflect rhythmic fluctuations of neuronal ensembles between states of low and high excitability (Bishop, 1932). Earlier research focused on investigating oscillations independently and linking them to high-level cognitive processes (e.g., theta to memory). However, this approach has been critiqued, as the exact role of each rhythm likely depends on an interplay among brain region, underlying neuronal substrate, and task context (Buzsáki, 2006; Wang, 2010). Our work is based on the hypothesis that oscillations reflect low-level mechanisms that can be flexibly used to change the dynamics of neuronal populations, thereby providing the scaffolding for different stages of information processing deployed in various cognitive processes. Thus, oscillations set the state of the system, either enhancing or attenuating activity (Klimesch et al., 2007), and inhibiting or facilitating network formation (Singer, 1999; Varela et al., 2001). Here, we focus on oscillations in the delta-to-theta, alpha, and beta bands in the context of working memory.

Slow rhythms in the delta-to-theta bands have been proposed to govern the sampling of our surroundings by providing alternating phases of high and low neural excitability and thereby phases of high and low perceptual sensitivity (Fiebelkorn et al., 2013; Herbst and Landau, 2016; VanRullen, 2016; Helfrich et al., 2018, 2019; Fiebelkorn and Kastner, 2019; Plöchl et al., 2021). Accordingly, sensory processing (and subsequent behavior) depends on the temporal coincidence of task-relevant targets with the oscillatory phase (i.e., when the phase with high excitability coincides with target occurrence, neural and behavioral responses are enhanced; Busch et al., 2009; VanRullen et al., 2011; Landau and Fries, 2012; Fiebelkorn et al., 2013; Zion Golumbic et al., 2013; Dugué et al., 2015; Henry et al., 2016). It has been shown that slow oscillations also control the timing of faster oscillations through phase–amplitude coupling (PAC; Canolty et al., 2010; van der Meij et al., 2012; Sauseng et al., 2019). However, it remains unclear how these cross-frequency interactions shape sensory processing and subsequent behavior. Here, we will investigate how the phase of slow rhythms, as well as the coupling between slow and faster rhythms, correlates with behavior.

The alpha rhythm is thought to reflect functional inhibition (Klimesch et al., 2007), such that increased alpha power suppresses processing in task-irrelevant regions and networks (i.e., increased alpha rhythm reflects reduced neural excitability), while decreased alpha rhythm facilitates processing in task-relevant ones, effectively gating the information flow (Jensen and Mazaheri, 2010). Accordingly, it has been demonstrated that working memory performance correlates positively with alpha activity in task-irrelevant regions (Haegens et al., 2010; Yu et al., 2017) and negatively in task-relevant regions (Jiang et al., 2015; van Ede et al., 2017), respectively. However, it remains unclear how alpha gating modulates internal sensory representations (as indexed by decoding performance), with studies reporting positive (i.e., decoding performance increases as alpha power increases; Kayser et al., 2016), negative (Barne et al., 2020), and null relationships (Griffiths et al., 2019). Here we will examine how alpha power correlates with working memory performance as well as the decodability of task features.

While the beta rhythm was traditionally regarded as a somatomotor rhythm (Pfurtscheller and Lopes da Silva, 1999), recent evidence suggests that beta is involved in top-down processing (Buschman and Miller, 2007) and long-range communication (Engel and Fries, 2010). However, it is unclear onto which low-level mechanisms beta oscillations map. One proposal is that beta, like alpha, gates information processing through inhibition (Lundqvist et al., 2016; Miller et al., 2018). A more recent proposal is that beta facilitates the endogenous activation/reactivation of cortical content representations (e.g., during working memory; Spitzer and Haegens, 2017). In this framework, beta band synchronization supports the endogenous transition from latent to active cortical representations during working memory, thereby providing a readout of the information held in memory. This proposal builds primarily on evidence from nonhuman primate research (Salazar et al., 2012; Haegens et al., 2017; Bahmani et al., 2018) and is yet to be explicitly tested in the human brain (Herding et al., 2016). Here we will test the involvement of beta oscillations in circuit activation/reactivation, the possibility of the existence of multiple betas, and how these tentative different betas correlate with behavior.

Combined, we propose a framework of oscillations as building blocks, in which delta-to-theta rhythms sample task-relevant input, while the alpha rhythm suppresses irrelevant input. Crucially, the beta rhythm recruits task-relevant circuits that maintain information in working memory. Finally, the timing of faster oscillations is controlled by slower rhythms via phase–amplitude coupling. Critically, this framework allows us to test specific hypotheses regarding how oscillations (separately and combined) orchestrate the functional architecture of information processing (and thereby shape behavior). In addition, it allows us to elucidate unsettled oscillatory characteristics (including the relationship of alpha to sensory representations and phase–amplitude coupling to behavior). For that purpose, we recorded MEG in healthy participants performing a visual delayed match-to-sample working memory paradigm with precueing and retro-cueing.

## Materials and Methods

### Participants

Participants were 33 healthy right-handed adults (21 female, 12 male; mean age, 24.6 years; age range, 20–33 years) without neurologic or psychiatric disorders, who reported normal hearing and normal or corrected-to-normal vision. The study was approved by the local ethics committee (CMO 2014/288 “Imaging Human Cognition”) and in accordance with the Declaration of Helsinki. Participants gave written informed consent and were remunerated for their participation.

### Visual working memory task

**Figure 1.**
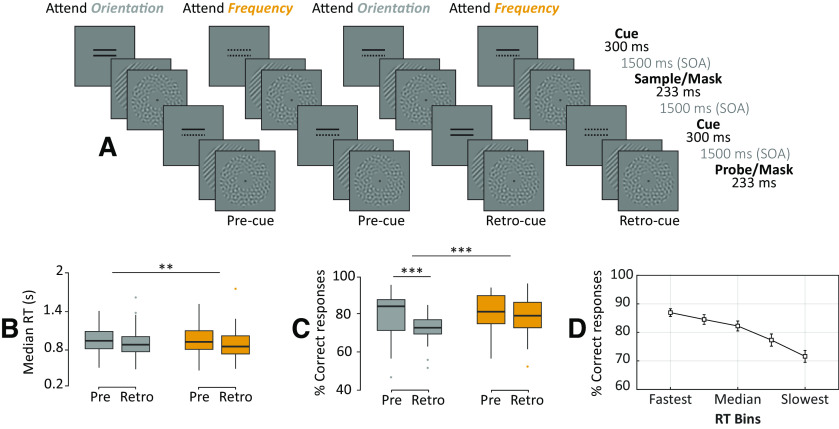
Paradigm and behavioral results. ***A***, Schematic of the four trial types: attend orientation (two solid lines) or attend frequency (two dotted lines) for precue and retro-cue conditions (first vs second cue is informative; uninformative cues consist of a solid and dotted lines). ***B***, ***C***, Participants were slower (***B***) and more accurate (***C***) matching the spatial frequency (gray bars) of the visual gratings compared with orientation (orange). This effect was mainly driven by the precue condition. Within each boxplot, the horizontal line represents the median, the box delineates the area between the first and third quartiles (interquartile range). ***D***, Average accuracy across RT bins. Error bars represent SEM. **p <* *0.01, ***p <* *0.001.

We used a single-item delayed match-to-sample working memory task where participants were instructed to compare sample and probe stimuli and to indicate whether the cued feature was the same or different between them (Fig. 1). Each trial contained the following four main events: a visual cue followed by a visual sample, a second visual cue, and a probe. All events were presented at a fixed stimulus onset asynchrony (SOA) of 1.5 s. The intertrial interval duration was jittered between 2 and 2.4 s.

We used a two (relevant feature: orientation vs frequency) by two (informative cue: precure vs retro-cue) factorial design. Participants were cued to compare sample and probe orientation in half of the trials, and spatial frequency in the other half. They were informed about the relevant feature by an informative cue, which occurred either before (pre; 50%) or after (retro; 50%) the sample presentation. These four trial types were randomly interleaved such that within each block participants performed all task conditions. Participants gave their response (“same” or “different”) by pressing a button with their right index or middle finger. The response button mapping changed on a block-by-block basis to avoid motor preparation confounds in our decision-related analyses.

Participants first performed a few short practice sequences of the task (20 trials each) in which feedback was provided on a trial-by-trial basis, with green and red fixation dots for correct and incorrect responses, respectively. During the main experiment, we recorded MEG while participants performed eight blocks of 64 trials each, in which feedback was provided on a block-by-block basis. The full recording session lasted ∼90 min.

### Stimuli

A bull’s-eye (outer black ring = 0.5° × 0.5° of visual angle; innermost black dot = 0.25° × 0.25° of visual angle) was presented at the center of the screen as the fixation point. Participants were instructed to always maintain fixation, and not to blink during the presentation of the stimuli. Each trial started with a cue presented for 300 ms. Next, a sample consisting of an oriented grating (Michelson contrast, 40%; spatial frequency, 1 cycle/degree; orientation, 45° clockwise or counterclockwise relative to vertical, randomized spatial phase) and a backward mask (a bandpass-filtered noise patch with matched contrast and spatial frequency as the grating) were presented sequentially for 230 ms. Both the grating and the mask were shown in an annulus (inner radius, 1.5°; outer radius, 7.5°; contrast of the stimuli decreased linearly to 0 over the outer and inner 0.5° radius of the annulus) around the central fixation (Fig. 1*A*). After an SOA of 1.5 s, a second cue was presented for 300 ms. The trial presentation finished with the probe presentation, which, similar to the sample, consisted of an oriented grating and a backward mask. The cued feature of the probe matched with that of the sample grating in 50% of the trials. The experiment was programmed with Psychtoolbox (Brainard, 1997) in MATLAB (MathWorks).

### Data acquisition

Stimuli were displayed on a semitranslucent screen (resolution, 1920 × 1080 pixel; refresh rate, 120 Hz) back-projected by a PROpixx projector (VPixx Technologies) during MEG recordings. Whole-head MEG data were acquired at a sampling frequency of 1200 Hz with a 275-channel MEG system with axial gradiometers (CTF MEG Systems, VSM MedTech Ltd.) in a magnetically shielded room. Six permanently faulty channels were disabled during the recordings, leaving 269 recorded MEG channels. Three fiducial coils were placed at the participant’s nasion and both ear canals to provide online monitoring of ] the participant’s head position (Stolk et al., 2013) and offline anatomic landmarks for coregistration. Eye position was recorded using an eye tracker (EyeLink, SR Research). Upon completion of the MEG session, the participant’s head shape and the location of the three fiducial coils were digitized using a 3D tracking device (Polhemus). Anatomical T1-weighted MRIs were obtained during a separate session. To improve coregistration of the MRIs and MEG data, earplugs with a drop of Vitamin E were placed at the participant’s ear canals during MRI acquisition. These anatomic scans were used for source reconstruction of the MEG signals.

### Behavioral analysis

The influence of cue condition (two levels: precue and retro-cue) and feature (two levels: orientation and spatial frequency) on median reaction time (RT; including correct responses only) and accuracy (percentage of correct responses) was tested using a linear mixed-effects model using the lme4 package (Bates et al., 2015) for R (R Foundation for Statistical Computing, 2014; http://www.R-Project.Org). For *post hoc* analysis, we used the Lsmean package (Searle et al., 1980) where *p*-values were considered as significant at *p *<* *0.05 and were adjusted for the number of comparisons performed (Tukey’s method).

### MEG preprocessing

MEG data were preprocessed offline and analyzed using the FieldTrip toolbox (Oostenveld et al., 2011) and custom-built MATLAB scripts. The MEG signal was epoched based on the onset of the first cue (*t* = −1 to 7 s). The data were downsampled to a sampling frequency of 300 Hz after applying a notch filter to remove line noise and harmonics (50, 100, and 150 Hz). Bad channels and trials were rejected via visual inspection before independent-component analysis (Jung et al., 2001) was applied. Subsequently, components representing eye-related and heart-related artifacts were projected out of the data (on average, four components were removed per participant). Finally, outlier trials of extreme variance were removed. This resulted in an average of 393 (±9 SEM) trials and 268 channels per participant for the reported analyses.

### Event-related fields

Single-trial data were baseline corrected (*t* = −0.1 to 0 s) before calculation of first-cue and grating-locked event-related fields (ERFs). In addition, a planar representation of the MEG field distribution was calculated from the averaged data (ERFs) using the nearest-neighbor method. This transformation makes interpretation of the sensor level data easier as the signal amplitude is typically maximal above a source.

**Figure 2.**
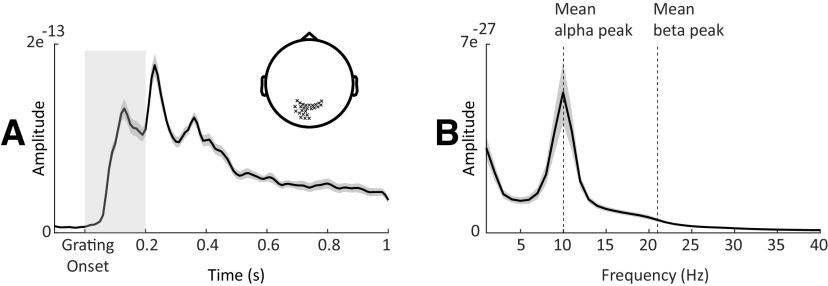
Alpha and beta peak detection. ***A***, Grand-averaged ERF of sensors (highlighted in sensor space) displaying maximal postgrating ERFs within 200 ms following the grating onset. Gray shaded area around the curve denotes between-participants SE. Light gray shaded box highlights the time period used to select channels with maximal postgrating ERFs. ***B***, Power spectra (averaged over same sensors marked in ***A***; *t* = −1 to 0 s relative to first cue onset) showing alpha and beta peaks.

### Spectral analysis

Our analysis aimed to highlight (1) the differential impacts of precueing and retro-cueing on the oscillatory dynamics of interest and (2) the behavioral relevance of these dynamics. For both of these contrasts, our spectral measures included delta-to-theta intertrial phase coherence, alpha power, and beta power. For the cueing contrast, spectral measures were compared between precue and retro-cue trials (including correct response trials only). For the behavioral contrast, trials from all conditions (including both correct and incorrect response trials) were pooled together and divided into five quantile bins (∼80 trials/bin) according to RT. Spectral measures were contrasted between the slowest and fastest RT bins to maximize statistical power.

For the 20 MEG channels displaying the maximal postgrating ERFs (channels selected individually per particpiant; Fig. 2), we extracted 1 s prestimulus windows (*t* = −1 to 0 s), multiplied these with a Hanning taper, and computed the power spectra (1–30 Hz; resolution, 1 Hz) using a fast Fourier transform approach. To determine the individual alpha peak frequency, we detected the highest local maximum within the 7–14 Hz band. To detect the beta peak frequency, linear regression (least-squares fit) was used to fit a linear model to the log-transformed spectrum in the beta range to compensate for the 1/*f* effect. The fitted linear trend was then subtracted from the spectrum, allowing for a more reliable beta peak frequency estimate within the 15–30 Hz band (Haegens et al., 2014).

We identified distinct alpha (mean ± SEM, 10.2 ± 0.2 Hz) and beta (mean ± SEM, 20.4 ± 0.3 Hz) peak frequencies for all participants. It is important to note that our peak estimations did not significantly vary when removing the 1/*f* background noise (alpha, *p* = 0.1; beta, *p* = 0.3). It is important to note that our peak estimations did not significantly vary when removing the 1/*f* background noise (alpha, *p* = 0.1; beta, *p* = 0.3). To create peak-centered signals, we computed time–frequency representations (TFRs) of the power spectra for the full trials per experimental condition. To this end, we used an adaptive sliding time window of five cycles in length per frequency (Δ*t* = 5/*f*; 20 ms step size), and estimated power after applying a Hanning taper. We created alpha-peak (individualized) and beta-peak (individualized) centered time-resolved signals with spectral bandwidths of 2 and 4 Hz, respectively. Power normalization was performed relative to a baseline (*t* = −0.4 to −0.2 s) for the cueing contrast and relative to the average of all RT bins for the RT contrast.

We estimated intertrial phase coherence (Tallon-Baudry et al., 1996) across trials, for each time point, frequency, and sensor, for each RT bin separately. This measure reflects the degree to which the phase of each frequency is aligned across trials. We averaged this measure between 1 and 6 Hz to reduce data dimensionality. In addition, we used the Tensorpac toolbox (Combrisson et al., 2020) to compute a time-resolved measure of PAC on source level. This algorithm measures PAC across trials (rather than across time).

### Statistical analysis

To investigate whether differences between conditions of interest (cueing contrast, precue vs retro-cue; RT contrast, slowest vs fastest RT bin) were statistically significant, we used nonparametric cluster-based permutation analysis (Maris and Oostenveld, 2007). In brief, this test first calculates paired *t* tests for each sensor at each time and/or frequency point, which are then thresholded at *p *<* *0.05 and clustered on the basis of temporal, spatial, and/or spectral adjacency. The sum of *t* values within each cluster is retained, and the procedure is repeated 1000 times on permuted data in which the condition assignment within each individual is randomized. On each permutation, the maximum sum is retained. Across all permutations, this yields a distribution of 1000 maximum cluster values. From this distribution, the probability of each empirically observed cluster statistic can be derived (evaluated at α = 0.05).

### Source reconstruction

For each participant, an anatomically realistic single-shell head model based on individual T1-weighted anatomic images was generated (Nolte, 2003). A grid with 0.5 cm resolution was created using an MNI template onto which the brain volume of each participant was morphed using nonlinear transformation. For each grid point, leadfields were computed with a reduced rank, which removes the sensitivity to the direction perpendicular to the surface of the volume conduction model. This procedure ensures that each grid point represents the same anatomic location across all participants.

To visualize the sources of our sensor-level spectrotemporal [power and intertrial coherence (ITC)] and temporal effects (ERFs), we used the frequency–domain adaptive spatial technique of dynamic imaging of coherent sources (DICS; Gross et al., 2001) and the linearly constrained minimum variance (LCMV) beamformer (Van Veen et al., 1997), respectively. Data from all conditions of interest were concatenated to compute the cross-spectral density (CSD) matrices (*t* = −1 to 2 s; multitaper method; Mitra and Pesaran, 1999) and the covariance matrices of the averaged single trials (*t* = −0.6 to 5.5 s; λ 5%) for the DICS and LCMV beamformer, respectively. Leadfields for all grid points along with the CSD/covariance matrices were used to compute a common spatial filter (i.e., common for all trials and conditions) that was used to estimate the spatial distribution of power/amplitude for our windows of interest. The source orientation was fixed to the dipole orientation with the highest strength.

Finally, the PAC analysis was performed on source level (i.e., virtual channels), for which the source space was subdivided into 22 anatomically defined brain parcels, including the intraparietal sulci, superior parietal lobes, frontal eye fields, middle frontal gyri, and the temporoparietal junction (Wallis et al., 2015). Sensor-level time series data were multiplied by spatial filters (constructed using LCMV beamformer) to obtain the time-resolved activity in each virtual channel.

### Multivariate pattern analysis

To investigate how oscillatory dynamics influence the information content of neural activity, we were interested in decoding two task features: (1) informative cue instruction (attend orientation or spatial frequency); and (2) sample (i.e., the first grating) properties (orientation, clockwise or counterclockwise; spatial frequency, low or high). For all multivariate pattern analysis, we used MNE Python (Gramfort et al., 2013) and Scikit-learn (Pedregosa et al., 2011). We implemented a fourfold cross-validation procedure within each participant. For each time point, we trained a logistic regression classifier (L2-regularized) on three folds and tested on the left-out fold. The analysis was shifted over time on a sample-by-sample basis. The input signal was the broadband signal low-pass filtered at 35 Hz (King et al., 2016; Gwilliams and King, 2020). We operationalized the decoding accuracy as the area under the curve (AUC) by evaluating the similarity between the true label categories of the test set and the probabilistic class labels (normalized distance from the fit hyperplane: “predict_proba” in scikit-learn) of the same trials. We computed the AUC under the null hypothesis by randomly shuffling the label categories and obtained a *p*-value as the proportion of the null AUC estimates that exceeded the true AUC (evaluated at α = 0.05).

**Figure 3.**
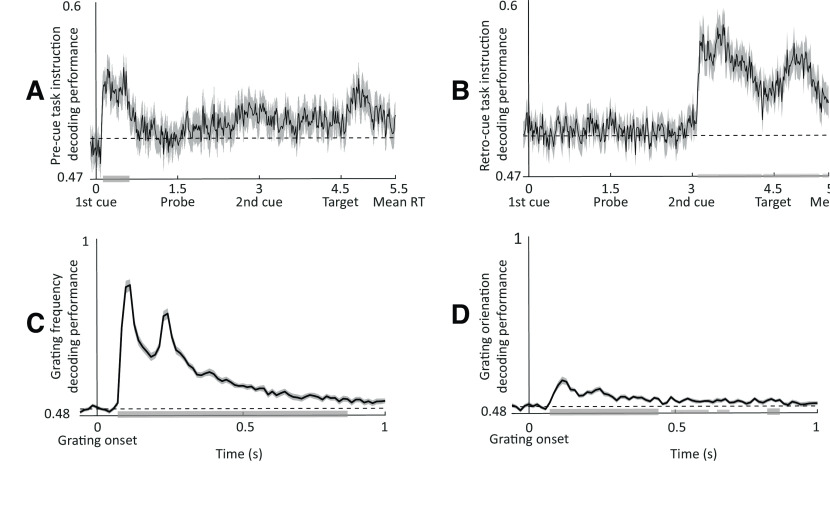
Decodability of task features. ***A***, ***B***, Temporal sample-by-sample decoding of cue features (attend frequency vs attend orientation) for precues (***A***) and retro-cues (***B***). ***C***, ***D***, Decoding performance for grating spatial frequency (low vs high; ***C***) and grating orientation (clockwise vs counterclockwise; ***D***). Gray bars indicate significance of decoding accuracy (*t* test vs chance).

We aimed to first demonstrate the decodability of the aforementioned features from the broadband time domain signal. We used trials from all conditions and tested decoding accuracy against chance level (i.e., AUC = 0.5 for binary classification) using nonparametric cluster-based permutation analysis (as described above). All task features could be decoded from the MEG signal: informative cue instruction (attend to frequency or orientation), grating orientation (clockwise or counterclockwise), and spatial frequency (low or high). For cue identity, group-level decoding performance peaked ∼150 ms after the onset of the first/second cue for the precue/retro-cue conditions, respectively (Fig. 3*A*,*B*), while, for grating properties, group-level decoding performance (for orientation and spatial frequency) peaked at ∼100 ms (Fig. 3*C*,*D*). It is important to note that the decodability of the grating features correlated negatively with reaction times (*r* = −0.5, *p* < 0.006) and positively with accuracy (*r* = 0.3, *p* = 0.042). This provides evidence that the decoded information reflects internal representation that is behaviorally relevant (Ritchie et al., 2019).

Next, we asked whether oscillatory state shapes neural stimulus representations as reflected by decoding performance. To test this, we evaluated how decoder accuracy was related to trial-by-trial fluctuations of prestimulus power. We used the classification procedure explained above, replacing the k-fold cross-validation with a leave-one-out cross-validation (LOOCV) procedure. In LOOCV, the classifier is fit to all trials but one, evaluating model performance on the remaining “left-out” trial as a single-item test set. This is advantageous because of the following: (1) it allows a maximal amount of data to be used for training, thus reducing noise in the model fit; and (2) it provides an unbiased single-trial decoding estimate, which can be analyzed by a binning approach. For each time point and test trial, we computed the probabilistic estimates of the logistic regression for our feature of interest (cue information) and then grouped these results into five bins relative to prestimulus occipital sensor theta, alpha, and beta power. The prestimulus window is *t* = −1 to 0 s for the first cue, *t* = 0.5–1.5 s for the sample, *t* = 2–3 s for the second cue, and *t* = 3.5–4.5 s for the probe. Afterward, for each frequency band separately, the time-resolved AUC signal was contrasted between the bins of lowest and highest power using nonparametric cluster-based permutation analysis.

Finally, to investigate the evolution of sample representations, we used the temporal generalization method (King and Dehaene, 2014). For this analysis, each classifier was trained on time *T* and tested on its ability to predict a given trial at time *T'*. This method estimates the similarity of the coding pattern at *T* and *T',* and thus the stability of the neural representation. To facilitate the interpretation of our results, we averaged the AUC for training times within the first 0.3 s following the sample onset. This was done separately for each bin and each band, followed by contrasting the averaged temporal generalization matrices between the bins of lowest and highest power using nonparametric cluster-based permutation analysis.

### Data availability

All data and code used for stimulus presentation and analysis are available online at the Donders Repository at https://doi.org/10.34973/tqy5-mh37.

## Results

### Behavioral results

To test our framework, we recorded MEG data from 33 healthy participants who performed a delayed match-to-sample working memory paradigm where they evaluated the orientation or spatial frequency of two visual gratings. The to-be-matched feature was indicated by a visual cue occurring either before (precue) or after (retro-cue) sample presentation.

Overall, participants were faster matching the orientation than the spatial frequency of the gratings (*F*_(1,32)_ = 11.2, *p* = 0.001; Fig. 1*B*). There was no significant difference in RTs between cue conditions (*F*_(1,32)_ = 2.7, *p* = 0.07) or significant interaction between feature and cue conditions (*F*_(1,128)_ = 12.7, *p* = 0.66). In terms of accuracy, participants were better at matching spatial frequency than orientation (*F*_(1,128)_ = 13.4, *p* < 0.001; Fig. 1*C*). There was no significant difference in accuracy between cue conditions (*F*_(1,128)_ = 2.7, *p* = 0.10), though there was a significant interaction between the cue and feature conditions (*F*_(1,128)_ = 12.7, *p* < 0.001): when matching the orientation of gratings, participants were better in the precue condition than the retro-cue condition (*t*_(32)_ = −3.7, *p* < 0.001), whereas when matching the spatial frequency of gratings, participants performed similarly in both cue conditions (*t*_(32)_ = 1.35, *p* = 0.17).

Note that the absence of main cue effects demonstrates that task difficulty (performance) was comparable across our conditions of interest (i.e., precue and retro-cue) and therefore does not constitute a confound in our subsequent analyses. Furthermore, accuracy linearly decreased with increasing RT (*R*^2^ = 0.21, *p* < 0.001; Fig. 1*D*), suggesting no speed–accuracy trade-off.

### Impact of cueing on oscillatory dynamics

To establish the proposed low-level mechanisms, we first investigated the differential impacts of precue and retro-cueing on oscillatory delta-to-theta phase, alpha power, and beta power. The critical difference between the pre-cue and retro-cue conditions is the time when participants are informed about the task-relevant/irrelevant feature. If alpha/beta decrease is critical for the inhibition of task-irrelevant features, we expect alpha/beta power decrease to be contingent on the informative cues, after which selection (i.e., inhibition of the task-irrelevant feature and/or enhancement of the relevant one) is possible. Similarly, if delta-to-theta phase plays a role in input sampling, we would expect an increase in phase alignment following the informative cues.

**Figure 4.**
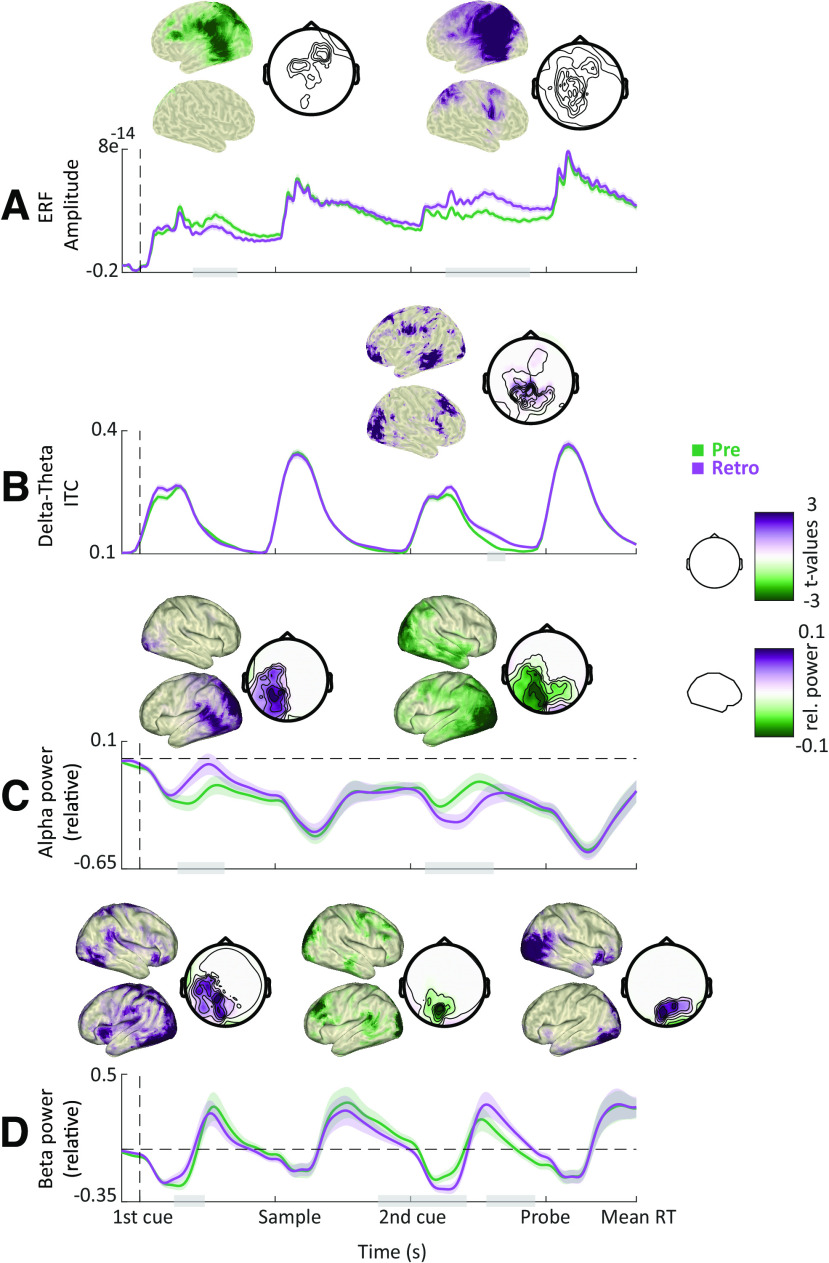
Cue-related power modulations. ***A***, Grand average ERFs for the precue (green) and retro-cue conditions (purple). Shaded areas denote between-participants SE. Gray bars indicate significant differences between conditions. Topographies show statistical and power distributions of these significant differences in sensor and source space, respectively. ***B***, Intertrial phase coherence (averaged between 1 and 6 Hz) for the precue and retro-cue conditions. ***C***, Time course of oscillatory power averaged within the alpha band. ***D***, Same as ***C*** but for the beta band. For all time courses, all sensors displaying significant differences as highlighted by the topographies were included in the plot.

Indeed, we found significant differences between precue and retro-cue conditions for all our frequency bands of interest (Fig. 4). Following the first cue, alpha and beta power decrease was stronger for the precue condition (in which the first cue was informative) compared with the retro-cue condition (Fig. 4*C*,*D*; cluster-based permutation test, *p* = 0.002 and *p* = 0.003; *t* = 0.35–1 s and 0.34–0.77 s; centered on occipitoparietal and temporal sensors). Differences between precue and retro-cue conditions were mainly localized to left and right occipital cortices, and right middle and superior temporal gyri.

This pattern was reversed following the second cue, with alpha and beta power decrease being stronger for the retro-cue condition (in which the second cue was informative) compared with the precue condition (Fig. 4*C*,*D*; *p* = 0.001; *t* = 3–4 s and 2.6–3.6 s; centered on occipitoparietal sensors). In addition, beta power decrease was stronger for the precue versus the retro-cue condition just before the probe presentation (Fig. 4*D*; *p* = 0.004; *t* = 3.8–4.4 s; centered on occipital sensors). Differences between precue and retro-cue conditions were mainly localized to left and right occipital cortices and the precentral gyrus, with beta effects extending more toward the dorsolateral prefrontal cortex.

Furthermore, following the second cue, delta-to-theta intertrial phase coherence was stronger for the retro-cue condition compared with the precue condition (Fig. 4*B*; *p* = 0.001; *t* = 3.6–4.1 s; centered on occipital, parietal, and temporal sensors). Differences between precue and retro-cue conditions were mainly localized to left and right occipital cortices, left inferior and middle temporal gyri, and left and right prefrontal cortices.

To summarize, the informative cue (i.e., the first cue on precue trials and the second cue on retro-cue trials) was followed by a stronger decrease in alpha and beta power, consistent with the functional inhibition account. In addition, the informative cue on retro-cue trials was followed by an increased phase alignment in the delta-to-theta band, consistent with a role in regulating input sampling.

### Impact of oscillatory dynamics on behavioral performance

Next, we asked whether any of the observed oscillatory dynamics impacted behavioral performance. We expected that faster responses would coincide with (1) an increase in the phase alignment of slow rhythms, reflecting their role in input sampling and temporal prediction; (2) a decrease in occipital alpha/beta power, reflecting their gating role; and (3) increased phase–amplitude coupling between slow and fast rhythms, reflecting the temporal coordination of fast rhythms by slower ones. We binned trials into five bins based on RTs and contrasted oscillatory dynamics between bins of the slowest and fastest RTs to test these predictions.

**Figure 5.**
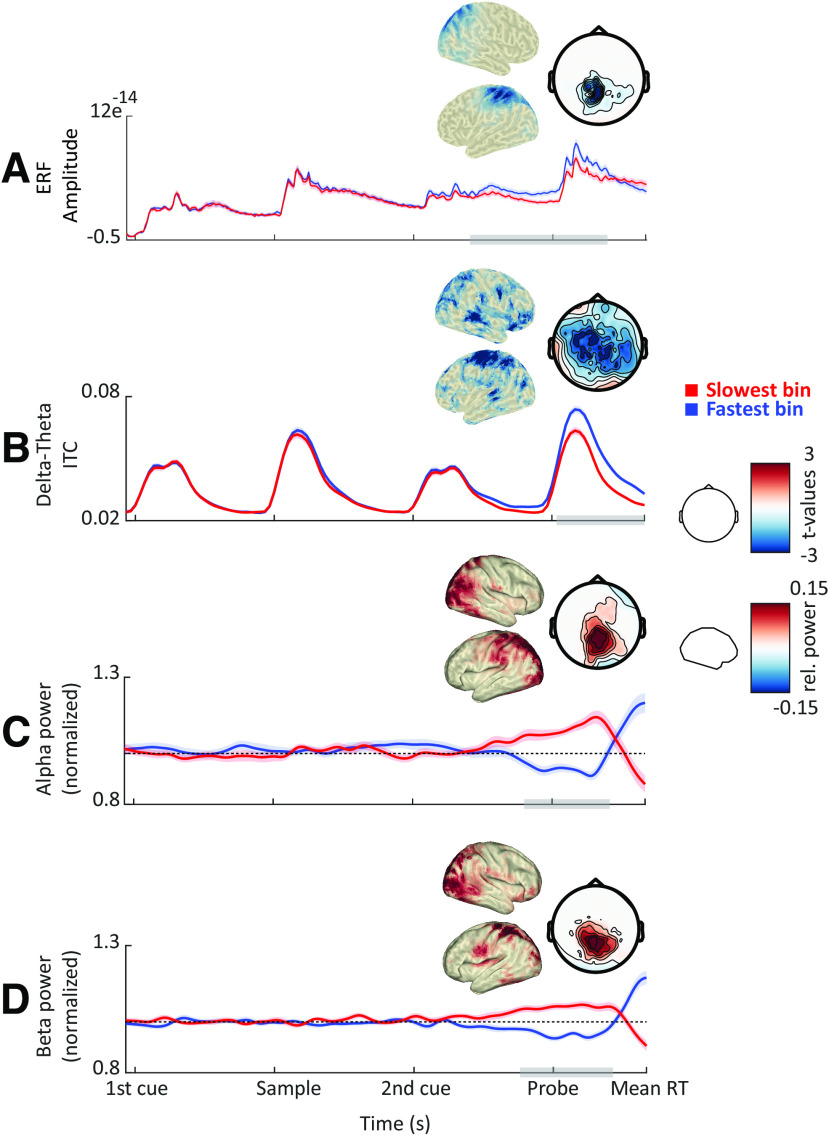
Correlates of behavioral performance: power and phase. ***A***, Grand average ERFs (occipital sensors) for the slowest (red) and fastest (blue) RT bins. Shaded areas denote between-participants SE. Gray bars indicate significant differences between conditions. Topographies show statistical and power distributions of these significant differences in sensor and source space, respectively. ***B***, Time course of intertrial phase coherence (averaged between 1 and 6 Hz) for the slowest and fastest RT bins. ***C***, Time course of oscillatory power averaged within the alpha band. Dashed lines represent mean power (i.e., normalized power = 1). ***D***, Same as ***C*** but for the beta band.

In line with our predictions, faster responses were associated with weaker alpha and beta power before and during probe presentation (Fig. 5*C*,*D*; *p* = 0.002 and *p* = 0.001; *t* = 4.1–5.1 s; centered on occipitoparietal sensors). These effects were localized to occipital cortices and the left inferior and parietal cortices. Furthermore, faster responses were accompanied by an increase in ITC in delta-to-theta frequencies (Fig. 5*B*; *p* < 0.001; *t* = 4.3–5.5 s; across widespread sensors). Differences were maximal in left and right postcentral and precentral gyri and lateral prefrontal cortices.

**Figure 6.**
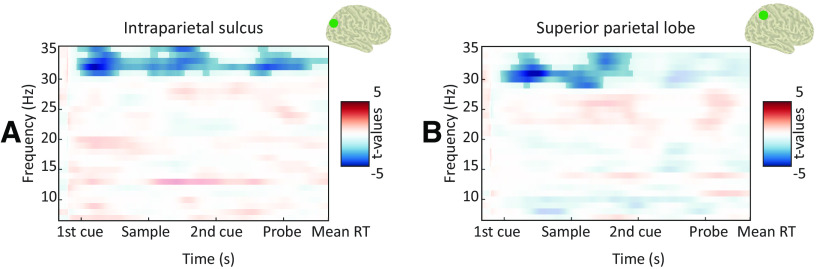
Correlates of behavioral performance: ITC and PAC. ***A***, TFR of statistical differences in PAC between the slowest and fastest RT bins (masked at *p* < 0.05) in the intraparietal sulcus (as highlighted in green on brain surface). Negative values (blue) indicate higher PAC for the fastest RT bin. ***B***, Same as ***A*** for the superior parietal lobe.

We found that the coupling between the phase of 1–3 Hz oscillations and the amplitude of 30–35 Hz oscillations was stronger for fast versus slow RTs in the intraparietal sulcus (Fig. 6*C*; *p* = 0.02; *t* = 0–5.5 s) and the superior parietal lobe (Fig. 6*D*; *p* = 0.07; *t* = 0–3 s). Combined, these results indicate that response speed was influenced by the phase alignment of delta-to-theta oscillations as well as phase–amplitude coupling between slow and fast rhythms.

In sum, a faster RT was preceded by (1) an increase in phase alignment and power of slow oscillations, consistent with a role in regulating input sampling; (2) a prominent occipitoparietal alpha/beta decrease, consistent with a gating-through-inhibition mechanism; and (3) an increased phase–amplitude coupling between slow and fast oscillations, consistent with a role of slow oscillations in temporal control of faster oscillations.

### Disentangling oscillatory dynamics from evoked responses

**Figure 7.**
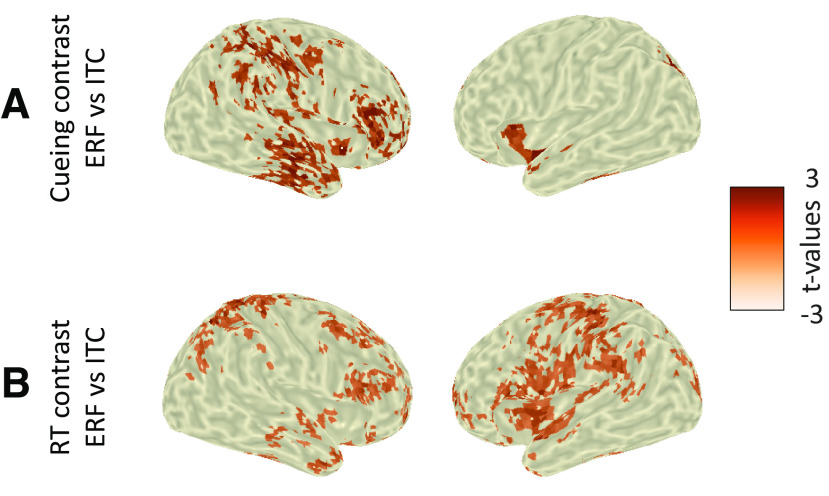
Correlates of behavioral performance: ERFs. ***A***, Source-level topographies of the statistical differences between ERF and ITC for the cueing contrast (precure vs retro-cue; *t* = 3.6–4.1 s; masked at *p* < 0.05). ***B***, Same as ***A*** for the RT contrast (slow vs fast RT bin; *t* = 4.4–5.1 s).

To ensure that the ITC results (for the cueing and RT contrasts) did not stem from low-frequency evoked (phase-locked) responses, we ran additional (control) analyses. For the cueing contrasts, we compared ERFs between cue conditions and found that following the first cue, ERF amplitude was higher for the precue compared with the retro-cue (Fig. 4*A*; *p* < 0.001; *t* = 0.6–1.1 s; centered on centroparietal sensors). In addition, following the second cue, ERF amplitude was higher for the retro-cue (Fig. 4*A*; *p* < 0.001; *t* = 3.4–4.5 s; widespread). Further, we compared the ERF and ITC differences between conditions at source level and found that ERF differences were more prominent in right precue and postcentral gyri and prefrontal cortex (Fig. 7*A*).

For the RT contrast, we compared ERFs between RT bins and found that ERF amplitude was higher for the fastest compared with the slowest RT bin (Fig. 5*A*; *p* < 0.001, *p* < 0.05; *t* = 3.7–5.5 s; centered on occipitoparietal sensors). Further, we compared the ERF and ITC differences between RT bins at source level and found that ERF differences were more prominent in left and right precue and postcentral gyri and prefrontal cortex (Fig. 7*B*). The significant differences between the localization of the ERF and ITC effects suggest that these effects (ERF and ITC) might reflect two separate phenomena.

Finally, we repeated the ITC analyses after subtraction of the evoked response from single-trial data. The results were similar to those of our initial analysis, as follows: for the cueing contrast, in the retro-cue condition, the presentation of the second cue was accompanied by an increase in ITC (*p* < 0.001; *t* = 3.5–4.3 s; widespread). For the RT contrast, faster responses were accompanied by an increase in ITC (*p* < 0.001; *t* = 4.7–5.4 s; widespread). The persistence of effects after the subtraction of evoked responses indicates that the low-frequency modulations cannot be fully explained by evoked activity.

### Impact of oscillatory dynamics on decoding performance

Finally, we investigated how oscillatory dynamics influence the information content of neural activity patterns. We expected this content, indexed by classification accuracy, to be boosted by a decrease in alpha power (i.e., release of inhibition) and an increase in beta power (reflecting circuit recruitment). We trained neural classifiers to decode the following two task features: (1) informative cue instruction (attend orientation or spatial frequency); and (2) grating properties (orientation, clockwise or counterclockwise; spatial frequency, low or high).

**Figure 8.**
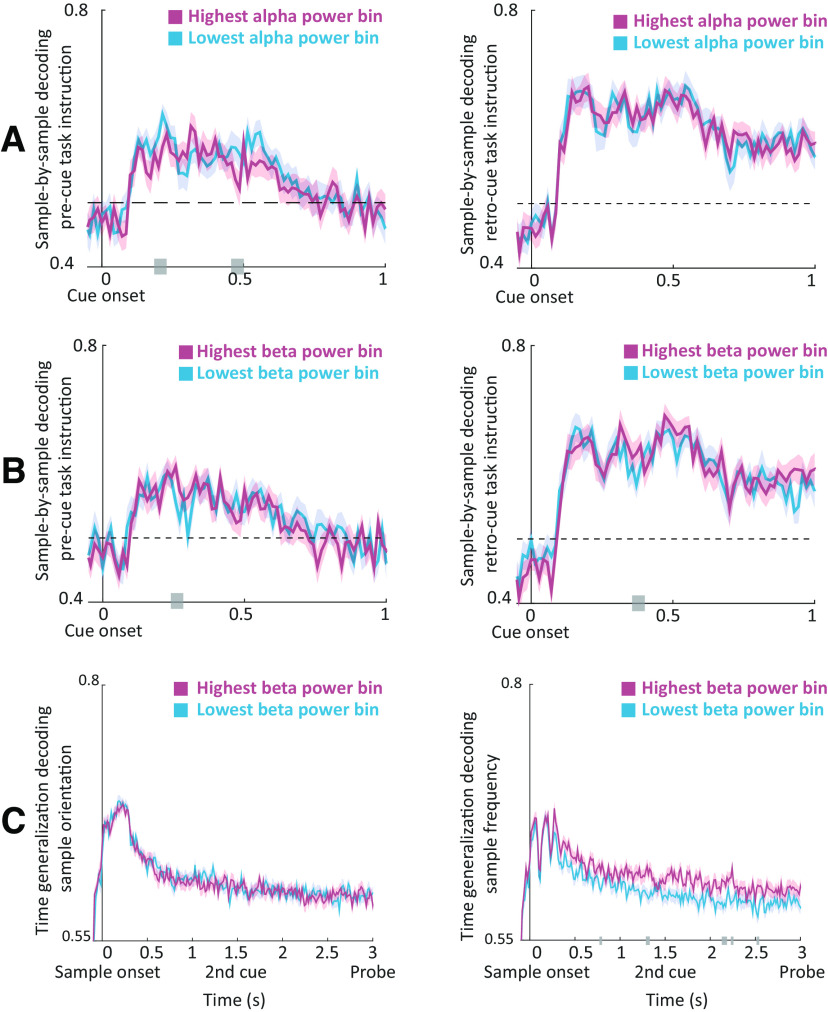
Effects of prestimulus oscillatory power on decodability. ***A***, Temporal sample-by-sample decoding of precue (left) and retro-cue (right) instruction for the bins of lowest and highest occipital alpha power preceding cue onset. ***B***, Same as ***A*** for the beta band. ***C***, Averaged time-generalization matrices (over the first 0.3 s of training time) of decoding the orientation of the sample (left) and frequency (right) for the bins of lowest and highest beta power preceding sample onset. Gray bars indicate significant differences between low-power and high-power bins.

Using a LOOCV procedure, we obtained unbiased single-trial responses of the classifiers. We then grouped these single-trial estimates into five bins relative to occipital theta, alpha, and beta power, and contrasted the decoding accuracy between bins of the lowest and highest precue power. In accordance with our alpha predictions, we found significant differences contrasting decoding accuracy between bins of low and high prestimulus occipital alpha power for precue instruction (Fig. 8*A*; *p* = 0.024; *t* = 0.2–0.21 and *t* = 0.47–0.49 s). In other words, decoding accuracy was higher for cues that were preceded by low alpha power. No significant effects were found for the decodability of retro-cue instruction by alpha power (*p* = 0.37).

In accordance with our beta predictions, we found significant differences contrasting decoding accuracy between bins of low and high beta power for precue instruction (Fig. 8*B*; *p* = 0.048; *t* = 0.26–0.27 s). In addition, we found a trend contrasting decoding accuracy between bins of low and high beta power for retro-cue instruction (*p* = 0.057; *t* = 0.37–0.39 s). Thus, contrary to alpha power, decoding accuracy was higher for cues that were preceded by high beta power. No significant modulations were found for the decodability of precue or retro-cue instruction by theta power (*p* > 0.5 and *p* = 0.21, respectively), suggesting that this modulation of neural stimulus information was specific to alpha and beta oscillations.

To further test our predictions regarding beta power and (reactivation of) information content, we focused on decoding the spatial frequency and orientation of the sample stimulus. To this end, we applied a time-generalization approach in which the classifier was trained at each time point and tested on all other time points, thus allowing us to test the temporal stability of the information content. This approach yields a two-dimensional matrix across training and testing times. To reduce dimensionality, we averaged decoding accuracy across the early training time points (0–0.3 s post-sample onset). We then contrasted the averaged decoding accuracy between the bins of lowest and highest prestimulus power.

In line with our predictions, we found significant differences between the averaged time-generalization matrices for decoding spatial frequency of the sample stimulus between bins of low and high occipital beta power (Fig. 8*C*; *p* = 0.022). In other words, in trials where the sample was preceded by high beta power, classifiers trained on early time points more accurately decoded the spatial frequency of the sample when tested on later time points (0.8–2.5 s) compared with trials preceded by low beta power. No significant effects were found for the decodability of the orientation of the sample (*p* = 0.47) based on beta binning; though, note that overall decoding performance was much lower for orientation than frequency (Fig. 3, compare *C*, *D*, overall decoding performance along the diagonal).

No significant effects were found for the decodability of the spatial frequency or orientation of the sample when binning was performed according to theta power (*p* = 0.11 and *p* = 0.2, respectively) or alpha power (*p* = 0.38, *p* = 0.46), suggesting that the maintenance of neural stimulus information was specifically supported by beta oscillations.

In summary, alpha and beta oscillations differentially modulated the information content of neural activity. We found a negative relationship between alpha power and cue representations, as predicted by the inhibitory gating account, while we found a positive relationship between beta power and cue and sample representations, as predicted by the circuit recruitment account.

## Discussion

In this study, we used a delayed match-to-sample working memory task with precue and retro-cueing to study a comprehensive framework that integrates the functional roles of specific neural oscillations, and, critically, the coordination between them. We directly tested our proposed framework in which slow rhythms sample task-relevant input, while alpha/beta gates the information flow by suppressing task-irrelevant processing, and beta further serves to recruit task-relevant circuits. Crucially, via phase–amplitude coupling, the timing of faster oscillations is controlled by slower rhythms. Indeed, in line with their role as a pace keeper of input sampling, we found that successful matching of probe and sample stimuli was preceded by an increased phase alignment that was confined to slow rhythms. As for the gatekeeping role of alpha oscillations, we demonstrated a pattern of alpha decrease that temporally tracked the presentation of the informative cue, correlated with faster behavioral performance and increased neural information content (indexed by decoding performance). Importantly, we shed new light on the dual role of beta oscillations: on the one hand, an alpha-like inhibitory role, gating the processing of relevant input, and on the other hand, a circuit-activation role, relevant for (reactivation of) information maintenance.

### Slow oscillations: the pace keeper

The first tenet of our framework is slow oscillations (1–6 Hz): we hypothesized that their phase regulates the sampling of task-relevant input and controls the timing of faster oscillations via phase–amplitude coupling. Indeed, we demonstrate that the intertrial coherence (i.e., phase alignment) of slow oscillations was more prominent after the presentation of the informative cues and was followed by faster responses. Interestingly, this effect occurred before probe onset and was maximal in the precentral and postcentral gyri and lateral prefrontal cortex. We also found that on fast trials, the phase of slow oscillations was more strongly coupled to the amplitude of faster oscillations (in the high beta range) in parietal areas.

We posit that this behavioral facilitation relies on more optimal temporal coordination reflected in the phase adjustment of slow oscillations and their coupling to faster oscillations, which, in turn, reflects better temporal prediction of probe onset. This is in line with previous reports that temporal predictions are associated with improved behavioral performance (Coull and Nobre, 1998; Miniussi et al., 1999; Nobre et al., 2007) and increased phase alignment of slow oscillations (Breska and Deouell, 2017), as well as recent evidence that the phase of slow oscillations predicts behavioral performance (Herbst and Obleser, 2019). This finding further corroborates previous reports of cross-frequency interactions during working memory (Bahramisharif et al., 2018; Bastos et al., 2018; Berger et al., 2019) and adds to a growing number of studies demonstrating the behavioral relevance of such coupling using intracranial (Axmacher et al., 2010) and scalp (Puszta et al., 2020) EEG. Thus, temporal coordination by slow oscillations governs the sampling of incoming stimuli (Busch et al., 2009; VanRullen et al., 2011; Landau and Fries, 2012; Fiebelkorn et al., 2013) and controls the timing of faster oscillations.

Finally, in addition to these delta-to-theta dynamics, we found that faster behavioral performance (and presentation of informative cues) co-occurred with stronger ERFs preceding and following probe onset [i.e., contingent magnetic variation (CMV), and N1/P2 and P3, respectively]. This ERF enhancement may reflect better temporal prediction of probe onset in fast trials, resulting in a more prominent anticipatory ramping up of neural activity (Brunia and van Boxtel, 2001; Bidet-Caulet et al., 2015; Breska and Deouell, 2017). In other words, this CMV amplitude increase in fast RT trials likely reflects increased motor preparation in anticipation of the probe onset (Gómez et al., 2007; Chennu et al., 2013; Bidet-Caulet et al., 2015).

One potential concern is that our spectral analysis picked up on this evoked activity (rather than genuine oscillatory dynamics). However, given that our phase results were not substantially altered by removing evoked activity and that delta-to-theta and ERF dynamics were not identically localized, we posit that these changes in evoked activity reflect an additional aspect of anticipatory mechanisms that are boosted by slow rhythm phase modulations (i.e., enhanced phase alignment leads to a stronger evoked response).

In summary, we outline how slow oscillations not only coordinate input sampling, but also faster oscillatory dynamics for optimal information processing and subsequent performance. Future research should investigate how this endogenous rhythmic sampling mechanism is deployed in contexts of fixed versus varying temporal structure.

### Alpha oscillations: the gatekeeper of information processing

We found that decreases in alpha power actively track the presentation of informative cues (i.e., regardless of its position in time, the informative cue was followed by a more prominent decrease in occipitoparietal alpha power). Our results are in line with the proposed gating role for alpha oscillations: once an input has been sampled through slow oscillations, its subsequent processing depends on its task relevance (i.e., alpha oscillations upregulate and downregulate cortical excitability in task-relevant and irrelevant networks, respectively; Klimesch et al., 2007; Jensen and Mazaheri, 2010). This regulation of cortical excitability has been shown to occur spontaneously (Linkenkaer-Hansen et al., 2004; van Dijk et al., 2008; Samaha et al., 2017; Iemi et al., 2021), or to track relevant input in space (Thut et al., 2006; Haegens et al., 2012; Mazaheri et al., 2014; ElShafei et al., 2018) or, as in the case of the current study, in time (Hanslmayr et al., 2011; Rohenkohl and Nobre, 2011; van Diepen et al., 2015; van Ede et al., 2017).

In addition, we found that faster responses were preceded by more prominent occipitoparietal alpha power decrease. This is in line with previous studies demonstrating that behavioral performance correlates negatively with alpha activity in task-relevant regions (Jiang et al., 2015; van Ede et al., 2017) and positively in task-irrelevant regions (Haegens et al., 2010; Yu et al., 2017). Both lines of results reflect the same low-level gating mechanism deployed to allocate more resources to task-relevant networks, either by directly boosting the processing of task-relevant information or by suppressing that of task-irrelevant information (i.e., distracting or competing stimuli).

Importantly, we demonstrate that prestimulus alpha power decrease enhances the neural decodability (i.e., sensory representations) of subsequent task-relevant items. These results are in line with previous work reporting a negative relationship between decoding performance and prestimulus alpha power (van Ede et al., 2018; Barne et al., 2020) and shows how alpha power, through regulating cortical excitability, might influence sensory representations.

### Beta oscillations: a dual role in information gating and circuit formation

We hypothesized that multiple beta mechanisms exist, with one beta mechanism playing a local functionally inhibitory role (“inhibitory beta”), similar to alpha, and another beta mechanism allowing top-down flexible activation of task-relevant circuits that maintain the information required to successfully perform the task at hand (“circuit beta”). These two mechanisms translate to opposite power modulations.

In support for its inhibitory role, we show that similar to alpha, decreases in prefrontal and occipitoparietal beta power are contingent on the occurrence of the informative cue and are associated with faster responses. This is in line with previous reports on the alpha-like gating role played by beta oscillations (van Ede et al., 2011; Bastos et al., 2018; Miller et al., 2018). Interestingly, while there is overlap in functionality and temporal patterns between alpha and beta, the localization of the inhibitory beta effects extends more toward frontoparietal regions, relative to that of the alpha effects.

In support for its circuit role, we demonstrate that increases (rather than decreases) in beta power index a stronger decoding and maintenance of task-relevant features (i.e., the decodability of stimuli preceded by high beta power was boosted and generalized for longer durations). This is in line with previous studies demonstrating that beta power correlates with stimulus information maintained in working memory as well as subsequent decision outcomes (Haegens et al., 2017; von Lautz et al., 2017; Proskovec et al., 2019). Here we go one step further, by directly linking the power of beta oscillations to the quality of information content in neural activity patterns. We posit that stronger beta synchronization indexes a stronger (longer lasting) activation of task-relevant circuits (i.e., working memory nodes). In turn, this enhanced circuit recruitment leads to more efficient maintenance of stimulus features, and thus allows them to be decoded for longer durations. These beta dynamics are in contrast with an alpha-like information-gating mechanism, which showed an inverse relationship with stimulus decodability.

Together, our results corroborate the proposed dual roles for beta rhythms in our framework (Spitzer and Haegens, 2017; Miller et al., 2018). Future invasive studies are required to dissociate the underlying generators (e.g., on the cell circuit/laminar level) of these proposed dual betas. In addition, the temporal and spatial evolution of alpha and beta rhythms should be thoroughly compared, to highlight potential differences and similarities in their roles in active inhibition.

### Conclusion

In summary, we demonstrate how rhythms from the delta to beta range might subserve higher-level cognitive functions by providing low-level mechanistic operations. Combined, these oscillatory building blocks allow for selective sampling of input, disengaging and engaging of task-irrelevant and relevant networks, and temporal organization of these respective dynamics. Critically, we find that these oscillatory dynamics correlate both with the behavioral performance of the participant, and with the information content in the recorded brain signal.

## Synthesis

Reviewing Editor: Nicholas J. Priebe, University of Texas at Austin Decisions are customarily a result of the Reviewing Editor and the peer reviewers coming together and discussing their recommendations until a consensus is reached. When revisions are invited, a fact-based synthesis statement explaining their decision and outlining what is needed to prepare a revision will be listed below. The following reviewer(s) agreed to reveal their identity: NONE.

The authors have adequately addressed the outstanding issues.
